# Challenges and Considerations of Preclinical Development for iPSC-Based Myogenic Cell Therapy

**DOI:** 10.3390/cells13070596

**Published:** 2024-03-29

**Authors:** Congshan Sun, Carlo Serra, Brianna Harley Kalicharan, Jeffrey Harding, Mahendra Rao

**Affiliations:** 1 Vita Therapeutics, Baltimore, MD 21043, USAmrao@vita-therapeutics.com (M.R.); 2 Department of Neurology, Baylor College of Medicine, Houston, TX 77030, USA

**Keywords:** iPSC, stem cell therapy, muscular dystrophy, preclinical studies, disease models, genetic modification

## Abstract

Cell therapies derived from induced pluripotent stem cells (iPSCs) offer a promising avenue in the field of regenerative medicine due to iPSCs’ expandability, immune compatibility, and pluripotent potential. An increasing number of preclinical and clinical trials have been carried out, exploring the application of iPSC-based therapies for challenging diseases, such as muscular dystrophies. The unique syncytial nature of skeletal muscle allows stem/progenitor cells to integrate, forming new myonuclei and restoring the expression of genes affected by myopathies. This characteristic makes genome-editing techniques especially attractive in these therapies. With genetic modification and iPSC lineage specification methodologies, immune-compatible healthy iPSC-derived muscle cells can be manufactured to reverse the progression of muscle diseases or facilitate tissue regeneration. Despite this exciting advancement, much of the development of iPSC-based therapies for muscle diseases and tissue regeneration is limited to academic settings, with no successful clinical translation reported. The unknown differentiation process in vivo, potential tumorigenicity, and epigenetic abnormality of transplanted cells are preventing their clinical application. In this review, we give an overview on preclinical development of iPSC-derived myogenic cell transplantation therapies including processes related to iPSC-derived myogenic cells such as differentiation, scaling-up, delivery, and cGMP compliance. And we discuss the potential challenges of each step of clinical translation. Additionally, preclinical model systems for testing myogenic cells intended for clinical applications are described.

## 1. Introduction

Human pluripotent stem cells (hPSCs) are cells that can self-renew and differentiate into the three primary cell lineages, which are the ectoderm, endoderm, and mesoderm. The natural form of pluripotent stem cells is that of embryonic stem cells (ESCs), which can be obtained from preimplantation embryos, while induced pluripotent stem cells (iPSCs) can be obtained from any of the somatic cell types. The ESC was discovered in the 1980s by cloning of cells from preimplantation embryos, and revolutionized developmental biology [1]. Induced pluripotent stem cells (iPSCs) were discovered by Yamanaka 2006 et al. by reprogramming murine somatic cells with four transcription factors, Oct3/4, Sox2, Klf4, and c-Myc, which perfectly circumvents the ethical issues of using ESCs as the source to derive therapeutic agents [2]. Subsequently, the same research group generated human iPSCs, revolutionizing the cell therapy field. Before the discovery of human iPSCs, stem cell therapy was focused on using primary somatic stem cells. The significant limitation of somatic stem cells in application is their lack of expandability and immune compatibility, which could both be resolved by using iPSCs.

hiPSCs were first generated by reprogramming human dermal fibroblasts with lentiviral vectors expressing Oct3/4, Sox2, KLF4, and c-MYC [3]. Other transcription factors were also identified to induce PSCs in combination with Oct3/4 and Sox2, such as Lin28 and Nanog [4]. Since the initial generation of the hiPSCs, efforts have been made to modify the reprogramming factor expression vectors as well as to optimize the methods of delivery to increase the efficiency and safety of the reprogramming. The first instrumental advancement was to design a single vector that contains all reprogramming factors instead of individual vectors. This reduces the harmful mutagenesis due to proviral sequence insertion [5]. Also, inserted lentiviral vectors were created that allow Cre recombinase excision to further increase the safety of hiPSCs [6]. The second advancement was made by using nonintegrating viruses like adenovirus or Sendai virus as reprogramming vehicles, which are diluted out of cells by passaging [7,8]. The current most utilized method is using a nonintegrating virus vehicle. Aside from the above two points, non-viral reprogramming methods including mRNA, miRNA, piggyBac, minicircle vectors, episomal plasmids, proteins, and small molecule compounds are all promising methods for reprogramming, but most of them suffer from low reprogramming efficiencies, high cost, or lack of supporting data [9]. The current trend in improving hiPSC reprogramming is focused on reducing insertional mutagenesis, which would lead to improved clinical safety, which also helps set the stage for application of the hiPSC in stem cell therapy.

To date, hiPSCs can be generated from practically any somatic cell type, and their induction has become a common procedure in both academic and industrial laboratories. As a result of this, induction reagents are becoming commercially available. In order to use hiPSCs to treat muscle-related disorders, a simple principle applies, which involves transplanting cells into patients to regenerate into healthy tissue to replace diseased tissue. Based on this, differentiating hiPSCs into healthy, functioning, and lineage-specified cells is necessary prior to transplantation. So far, although few in number, there have been clinical trials initiated for hiPSC cell therapies to treat spinal cord injury, Parkinson’s Disease, macular degeneration, retinitis pigmentosa, etc. [10]. However, there has been no record of trials using hiPSCs in muscular disease therapies.

**Figure 1:**
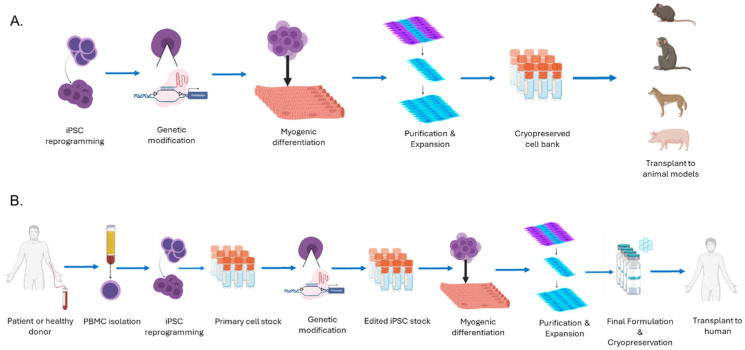
Flowchart of iPSC-derived myogenic cell production process for preclinical trials (**A**) and clinical trials (**B**). (**A**) In preclinical trials, cells obtained from a third party or in-house are reprogrammed into iPSCs and genetically modified (if applicable), according to the therapeutic requirements. The edited iPSCs are subject to myogenic differentiation, purification, and expansion. These are cryopreserved for preclinical trials in animal models. (**B**) In clinical trials, the manufacturing process needs to comply with appropriate FDA regulations. Cells that are obtained from a consenting donor (for example, blood cells) are reprogrammed into iPSCs. The iPSCs are cryopreserved as a primary cell stock (if applicable). These cells undergo genetic modification (if applicable), allowing for the creation of a secondary cell stock of edited iPSCs. The edited iPSCs are subject to myogenic differentiation, purification, and expansion. The expanded cells will be formulated into a final product and transplanted into clinical trial subjects. (Created with BioRender.com).

In this review, various challenges in each step of cell production (Figure 1) that have been preventing hiPSC application in treating muscle-related disease and potential solutions will be discussed. This includes common challenges in using hiPSCs, for example tumorigenicity, as well as specific challenges resulting from muscle being a solid tissue and the largest organ.

## 2. Differentiation and Maintenance of Myogenic Cells from Pluripotent Stem Cells

Human pluripotent stem cells (hPSCs) which include human embryonic stem cells (hESCs) and human induced pluripotent stem cells (hiPSCs) can self-renew and differentiate into the three primary cell lineages, which are the ectoderm, endoderm, and mesoderm. hESCs can be obtained from preimplantation embryos, while hiPSCs can be derived from any of the somatic cell types.

### 2.1. Myogenic Lineage Specification from hiPSCs

Since the discovery of hiPSCs, researchers have dedicated their efforts to differentiating these cells into specific somatic lineages to study tissue development and disease modeling, as well as for translational research drug screening and cell therapy. Differentiating hiPSCs involves replicating the internal and external developmental signaling cues during embryogenesis. Recently, the emphasis has shifted toward mimicking the embryonic environment through 3D cell cultures. Numerous protocols have been developed to guide the differentiation of hiPSCs into myogenic lineage cells, falling into the three main categories outlined below.

#### 2.1.1. Direct Programming

During embryonic development, myogenic transcription factors including Pax3 (paired box gene 3), Pax7 (paired box gene 7), and MyoD (myoblast determination protein 1) play critical roles in directing muscle lineage specification to form embryonic muscle tissue as well as residential satellite cells [11,12,13]. During skeletal muscle development, Pax3, Pax7, and MyoD bind to promoters and enhancers of myogenic genes, such as Myf5 (Myogenic factor 5) and myogenin, orchestrating remodeling of chromatin conformation and organization, thereby establishing epigenetic marks to determine myogenic cell fates [12,14,15]. As crucial as their functions are in myogenic lineage specification, each of these factors was reported to be able to induce myogenic lineage specification. Doxycycline-inducible MyoD was first expressed in mouse ESCs. Upon doxycycline treatment, mouse ESCs expressed MyoD, which subsequently led to expression of Pax7 from days 4 to 7. Overlapping with the MyoD and Pax7 expression from days 4 to 7, myotubes began forming and desmin was detected from day 5 [16]. Later, Abujarour et al., 2014 induced MyoD expression in hiPSCs for 4 days and showed myotube formation within 7 days from the beginning of the induction [17]. Doxycycline-inducible Pax3 and Pax7 (iPAX3 and iPAX7, respectively) were introduced into mouse ESC cells and later human ESCs and iPSCs [18,19]. This protocol consisted of embryoid body formation for 2 to 5 days followed by doxycycline induction of Pax3/7 expression for 6 to 9 days. At the end of the myogenic differentiation process, Pax3- or Pax7-expressing cells reached as high as 90%. When the cells were transplanted into immunodeficient and injured mice, cells were shown to participate in muscle regeneration, with a subset of transplanted cells homing to the local satellite cell niche area. Initially, the iPAX3 and iPAX7 were integrated into the genome using lentiviruses, leading to nonspecific integration. Due to the safety concerns relating to random genome integration, iPAX7 was then inserted into a genomic safe harbor. At the same time, strategies using minicircular DNA carrying PAX7 were reported; however, the efficiency of Pax7 induction was lower with minicircular DNA delivery [20,21]. More effective nonintegrating methods will need to be established to improve the safety and effectiveness of the direct programming method.

#### 2.1.2. Transgene-Free 2D Myogenic Differentiation

Chemically based myogenic lineage specification was introduced because it avoids the use of external genetic material that causes integration mutations; hence, it is more suitable for clinical application. During embryonic myogenesis, the paraxial mesoderm forms first, which develops into the presomitic mesoderm and later on a myotome. The Wnt signaling pathway was identified as one of the most important signaling pathways in the development of the paraxial mesoderm [22]. Based on this, researchers found that activating Wnt signaling alone in ESCs with the GSK3 (glycogen synthase kinase 3) inhibitor CHIR99021 could induce the formation of the paraxial mesoderm marked by MSGN1 (Mesogenin 1) and TBX6 (T-Box Transcription Factor 6) expression [23]. To further induce PAX3+ presomitic mesoderm commitment, BMP (bone morphogenetic protein) signaling inhibition by LDN193189 [24] or NOTCH signaling inhibition by DAPT [25] was found necessary. Following PAX3 induction, cells were cultured for ≥20 days to induce the formation of MyHC (myosin heavy chain)-positive myotubes, PAX7+ myogenic progenitor cells, and MYOD+ and/or myogenin+ myoblasts. With the myogenic lineage specification by WNT activation and BMP/NOTCH inhibition established, researchers worked on modifying the protocol to achieve the desired cell population with improved regeneration potential. Addition of the TGF-β (transforming growth factor beta) signaling inhibitor SB431542 and growth factors such as FGF2 (fibroblast growth factor 2), as well as prolonged culturing, were demonstrated to enhance myogenic lineage specification and cell maturation [26,27,28]. Despite the advantages chemically defined lineage specification holds toward being applied in clinical settings, the long period of culture and the heterogeneous cell types present in the culture remain challenging for mass production.

#### 2.1.3. Three-Dimensional Myogenic Differentiation

It is well known that, when cultured in a low adherent vessel, hiPSCs aggregate and spontaneously differentiate into three-dimensional embryonic bodies that contain three germ layers. If lineage induction reagents are added, the 3D spheroid can differentiate toward specific cell lineages which can represent the target tissue. The 3D in vitro tissue model, which is also called an organoid, has been generated for a variety of tissues, e.g., brain and intestine. However, few organoids have been established for muscle tissue. The process for generating a muscle organoid, like 2D lineage myogenic specification, recapitulates the development of the paraxial mesoderm and the embryonic myotome. Self-organized embryoid bodies formed from iPSCs are either embedded in an extracellular matrix or kept floating in a non-adherent culture vessel. In the culture, a WNT activator and BMP inhibitor are added for the first 3 to 5 days, allowing formation of the neuroectodermal lineage. When skeletal muscle is the only tissue required in the organoid, FGF2, HGF (hepatocyte growth factor), and IGF (insulin growth factor) are used to induce the dermomyotome [29,30,31]. If the formation of a neuronal muscular junction is desired, retinoid acid, Wnt1a, and Sonic hedgehog could be added to induce the neural tube following the neuromesoderm induction prior to the dermomyotome induction [32]. The advantage of differentiating using a muscle organoid is that the myogenic progenitor cells that grow in the organoids are more developmentally mature compared with those in 2D lineage specification, which could be due to the organized muscle fiber and neuron formation [30]. More recently, one study spiked iPSCs with embryonic fibroblasts and endothelial cells to generate an embryoid body which could generate myogenic progenitor cells more efficiently [33]. Although there are some advances being made and some protocols are available, more efforts are needed to improve the efficiency of muscle organoid formation and downstream myogenic cell purification.

### 2.2. Lineage Specification Efficiency, Purification Process, and Tumorigenicity

The generation of large quantities of myogenic progenitors which could become muscle stem cells upon transplantation is critical to muscle cell replacement therapy efficiency. During the myogenic lineage specification process in vivo, the paraxial mesoderm is referred to as the presomitic mesoderm which consists of a posterior and anterior region. The posterior region is formed from neuromesodermal progenitors and could form either neural or mesodermal cells depending on Fgf8 and Wnt3a signaling, respectively. The anterior region forms somite which could differentiate into dermomyotome giving rise to skeletal muscle [34]. The nature of the paraxial mesoderm as having both neuronal and mesodermal origin causes the mixed cell types present in the chemically defined in vitro myogenic lineage specification culture. With different lineage specification methods, the cell type compositions are different. The most common cell types appearing have been neuronal cells and mesenchymal cells [35]. When a cell transplantation experiment was performed, engraftment efficiency of the muscle cell population was greatly compromised by the contaminant cells [36]. The impure cell population also raises safety concerns. All in all, selecting the myogenic cells’ post-myogenic lineage specification process is crucial to the generation of a cell product that will regenerate into muscle tissue. So far, fluorescence-activated cell sorting (FACS) has been mainly used as the purification method [37]. As each lineage specification method induces the lineage specification in a slight different manner, the surface protein presentations are different (Table 1). Several surface markers have been identified to enrich the myogenic progenitor population that have been reported to possess superior myogenic regeneration capabilities as measured by in vitro myotube fusion and in vivo engraftment.

When it comes to isolating desired cell populations, FACS and magnetic-activated cell sorting (MACS) are usually considered [41]. FACS, which is used in research settings, could select cells more precisely with gating; however, it lacks scalability due to its limited sorting speed (~5 million cells per hour) [42]. MACS, which has the capacity to process 100 million cells within thirty minutes, is, in comparison, a more viable option for purifying cells for clinical application, despite the lower resolution of cell selection compared to FACS. The feature of quick processing time in MACS especially accommodates the muscle cell replacement therapy partly because muscle is the largest organ of the body and it requires a large number of cells (millions to billions) to generate enough volume of muscle tissue to achieve functional rescue from muscle-related disorders [43]. While considering speed and volume, attention also needs to be paid to purity, as residual iPSCs or partially differentiated iPSCs could lead to tumorigenicity, which mainly includes teratoma formation and oncogene c-MYC expression-induced tumor formation [44]. Apart from using surface markers to isolate myogenic populations, it is critical to confirm if any iPSCs or partially differentiated iPSCs remain in the cell product [45]. Flow cytometry to detect pluripotency surface markers like SSEA4 and TRA-1-60 and quantitative PCR to detect gene expression of pluripotent genes like OCT4, Lin28, and ESRG have been employed to verify the cell product [46,47]. The caveat of selecting the appropriate iPSC residual assay is that due to different differentiation processes inducing different sets of gene expression, specific detection methods and marker selection are required for each cell therapy product.

### 2.3. Maintaining Muscle Progenitor Cell Characteristics in Culture

The existing myoblast transplantation clinical trials were mostly carried out for Duchenne muscular dystrophy (DMD) via intramuscular transplantation [48,49,50,51,52,53,54,55,56]. Even for a small surface area of 25 mm^2^, 100 million cells were needed for the trial. When it comes to the large volume of muscle, billions of cells are needed in order to restore muscle function. Extensive cell expansion is required to produce a large number of cells. As was mentioned earlier, myogenic progenitor cells are the desired population for cell therapy application; therefore, maintaining the progenitor characteristics in culture is critical. Muscle progenitor cells, which include the satellite cells, have the tendency to differentiate into myocytes and fuse into myotubes when cultured in a nutrient-thin environment. DMEM F10 medium supplemented with serum and chicken embryonic extract was traditionally used to clonally expand human satellite cells [57]. This medium is known to support cell proliferation and slow down spontaneous differentiation. However, as clinical application requires a chemically defined medium, studies were carried out to search for a replacement. The combination of FGF, epidermal growth factor (EGF), and insulin in a culture medium for human satellite cells was reported to have the same effect as chicken embryonic extract. Also, the combination of insulin, EGF, dexamethasone, bovine serum albumin (BSA), and bovine fetuin could replace FBS [58].

It has been reported that satellite cells can retain their stemness in the stem cell niche environment, but when the cells are expanded in vitro they progressively lose their stemness. The niche environment, which consists of chemical and physical cues, plays a critical role in maintaining stemness. Among the chemical cues, NOTCH signaling, which functions through Delta-1, was reported to be the major regulator of satellite cell proliferation and self-renewal [59,60]. Another important pathway is the p38 MAPK pathway, which regulate satellite cell differentiation through activating MyoD [61]. Other chemical cues such as growth factors and cytokines such as basic fibroblast growth factor (bFGF), insulin growth factor (IGF), epidermal growth factor (EGF), brain-derived neurotrophic factor (BDNF), vascular endothelial growth factor (VEGF), platelet-derived growth factor (PDGF), tumor necrosis factor-like weak inducer of apoptosis (TWEAK), interleukin 6 (IL-6), and leukemia inhibitory factor (LIF) are well documented to regulate satellite cell proliferation and differentiation [62]. Supplementing these in the growth medium may effectively maintain or even increase the PAX7+ cell ratio. Based on the similarity of hiPSC-derived myogenic progenitors (hiPSC-MPCs) and satellite cells, the discovery made in the satellite cells can be translated into developing hiPSC-MPC medium formulation.

Regarding physical cues, mechanical sensing impacted by the stiffness and elasticity of the surface plays a significant role in satellite cell self-renewal and proliferation. It has been reported that softer surfaces, for example, soft fibrin gel, could result in selectively expanding satellite cells without losing Pax7 expression [63]. Researchers also attempted to reconstruct synthetic constructs that mimic the satellite cell’s native environment. For example, collagen-based hydrogel was constructed to be 1.33 ± 0.18 kPa to induce the quiescence of mouse satellite cells [64]. One contributing factor to mechanical sensing of satellite cells is hippo signaling, in which the localization of the downstream effectors Yap and Taz is regulated by stiffness. When Yap and Taz relocate to nuclei, they activate genes that regulate proliferation and differentiation of satellite cells [65,66,67]. Another application of physical cues to expand satellite cells was reported to use ice-cold treatment during satellite cell expansion to selectively enrich stem cells because they detach earlier than differentiating cells [68]. However, due to the high cost of manufacturing a culture surface with a different stiffness, it is unlikely that this could be applied to cell cultures on a large scale. More research in engineering is required to produce such surfaces.

## 3. Genetic Modification

### Genetic Modification Strategies

The term genetic engineering describes the approaches to modifying genomic DNA such as removing endogenous sequences or genes, suppressing or activating genes, or inserting novel sequences. Methods to insert transgenic DNA sequences have historically relied on random integration into the genome using viral vectors [69,70] or transposon-based plasmids [71]. Viral methods rely on the production of transgene-containing viral particles, which can be used to transduce the cells of interest to deliver transgene payloads as single- or double-stranded RNA/DNA sequences. Transposon methods consist of delivering transgene-containing plasmids, as well as transposase enzymes, that are integrated into the genome of the target cell [72]. There are important differences between these methods, including the potential payload size, the risk of immunogenicity, payload manufacturing (virus vs. plasmid), and genomic integration site biases [73]. Nevertheless, both approaches have been used to correct mutations or deficiencies in genes causing muscular dystrophies, as well as for delivery of the DMD or mini-DMD coding sequence [14,74,75,76], many studies on which have been reviewed elsewhere [77]. In fact, viral-vector-based gene correction has already entered into several clinical trials [78], and in 2023 the FDA approved one such therapy, ELEVIDYS, for children age 4–5 with DMD.

The discovery and invention of natural and artificial enzymes that can target specific genomic sequences, including meganucleases, zinc finger nucleases (ZFNs), transcription activator-like effector nucleases (TALENs), and CRISPR-Cas9, has revolutionized the field of genome engineering. In particular, CRISPR has allowed targeted genomic editing in a way that generally is simpler, cheaper, and more versatile than almost anything that has come before [79,80]. The most common use of CRISPR in gene editing relies on making targeted double-stranded breaks (DSBs) in the DNA with a Cas protein, such as Cas9, which can be used to create gene knock-outs or to insert transgenes near the DSB. The Cas9 protein is targeted to the genomic region of interest with the addition of a short guide RNA (gRNA) oligo that is complementary to the genomic sequence. Designing and acquiring gRNAs are relatively simple and fast processes. Moreover, additional classes of the CRISPR system are continually being discovered or engineered, which has increased the variety of edits that can be performed on DNA [81]. These include targeting mRNA instead of DNA with Cas13 [82], re-coding or adding individual nucleotides with base and prime editing [83,84], and activating or repressing transcription with Cas proteins fused to regulatory domains [85,86].

In autologous cell therapies relying on CRISPR, correcting the gene mutation or downstream transcriptional products in cells derived from muscular dystrophy patients is crucial to the therapeutic effect of the product. Generally, muscular dystrophy patients’ blood cells are obtained and reprogrammed into hiPSCs, which is followed by gene editing. Given the diversity of mutations involved in different muscular dystrophies, there are countless potential strategies for CRISPR-based correction, many of which are being explored. For instance, a simple CRISPR-induced DSB can be strategically made in a mutated dystrophin gene to restore expression of the protein via exon skipping [87] or reading frame correction [88]. Alternatively, CRISPR can be used to integrate specific DMD exons into the mutated DMD gene [89]. In principle, an entire mini-DMD coding sequence could be integrated into amenable genomic locations such as safe harbor sites or linked to the expression of endogenous genes. In cells derived from facioscapulohumeral muscular dystrophy (FSHD) patients, CRISPR can be used to ablate expression of the toxic DUX4 protein by deleting specific genomic sequences [90] or suppressing DUX4-encoding transcripts [91]. Of course, all of these methods rely on the delivery or expression of CRISPR-related proteins and gRNAs in the autologous cell. This can be achieved by methods including electroporation, lipid delivery, or viral and transposon-based vectors.

There are several challenges with CRISPR-based engineering of iPSCs on clinical application. Any designed gRNA has the potential to hit off-target sites in the genome [92]. Often, this problem can be mitigated by optimizing gRNA design and/or using high-fidelity Cas protein variants with reduced off-target activity [93]. Many assays, such as GuideSeq [94] and rhAmpSeq [95], have been developed for highly sensitive detection of off-target sites. In autologous therapies stemming from edited, clonally derived lines, the challenge of off-target detection is simplified by the lack of a population with heterogenous edits. Nonetheless, the off-target potential and profile of a given gRNA sequence remain relevant and major concerns for the FDA with any CRISPR-based gene editing. As a foreign protein, there is also the potential for an immune response to be induced after transplantation into a recipient [96]. The risk of immunogenicity will depend on how long the Cas protein is present in the cells, how it was delivered, and the genetic background of the host. Crucially, it will also depend on the editing strategy itself. For instance, in Cas9-induced gene knock-out or transgene knock-in, only transient expression is needed in the autologous cells ex vivo, which almost completely removes the risk of a downstream immune response. However, CRISPR interference approaches, such as those blocking DUX4 transcription in FSHD cells, require continual expression of the CRISPR components for the life of the cell.

The use of iPSC-derived cells comes with the inherent risk of contaminating iPSCs in the final cell product, which can be tumorigenic or interfere with the activity of the differentiated component. The use of nucleases during genome editing only increases the safety risk of these cells. Any DNA alteration, especially double-stranded breaks, increases the chance of unwanted chromosomal changes, such transversions, translocations, and large deletions, among many other outcomes. For these reasons, and especially for transplanted cells intended to survive long-term, it may be crucial to also equip the cells with safety or suicide systems [97], such as TK linked to endogenous CDK1 [98], or iCasp9 linked to *OCT4* [99], among many other invented systems. Depending on the design, and in case of an unwanted or aberrant outcome after engraftment, these systems can allow for the ablation of the transplanted cells or only the contaminating iPSC-derived component.

Ultimately, given the expanding and extremely versatile toolkit, the use of CRISPR gene editing in the treatment of muscular dystrophies will only continue to grow, especially for autologous, cell-based therapies.

## 4. Development and Challenges of Disease Models for Preclinical Studies

Muscular dystrophies are caused by genetic mutations that are largely heterogeneous. For example, DMD, which is the most common muscular dystrophy, was found to contain exon deletion (65%), duplications (10%), or smaller-scale mutations (25%) in different regions of the DMD gene [100,101,102]. Creating a disease model that is appropriate to represent all the variations in the genetic mutations of a muscular dystrophy remains challenging.

### 4.1. Cellular Models

Recent years have seen the robust development of cellular models for studying muscular dystrophies due to their cost efficiency, ease of manipulation, and the feasibility of culturing multiple cell lines carrying different genetic mutations, all of which overcome some limitations of animal models. The key to a successful cellular model is to recapitulate the pathological features, which include decreased muscle contraction and scarce regeneration. So far, researchers have developed and used cellular models such as primary myoblasts, iPSC-derived muscle cells, organoids, and muscle-on-a-chip models to study disease mechanisms and test therapies [103]. Most early cellular models focused on using primary myoblasts derived from patients in a 2D culture. For example, in DMD, the myoblasts, when fused into myotubes, do not express dystrophin. When considering using this as a model for iPSC-derived muscle cell therapy, co-culturing therapeutic cells and the disease cells can be one approach to evaluate the restoration of dystrophin expression. However, since the 2D model only provides a single outcome, it does not include other crucial features of the muscle tissue such as contractility and tissue regeneration which are indications of muscle health. Moreover, the iPSC-derived muscle cell therapy requires transplanting cells into the tissue, which simply cannot be replicated by a 2D culturing system. To overcome this, in the past 30 years, 3D tissue engineered skeletal muscle made with hydrogels, collagen, or fibrin has become the ideal candidate for in vitro modeling for myogenic cell transplantation therapies. It was reported that these engineered 3D muscle constructs could mimic the in vivo muscle bundles in terms of measurable contraction as well as satellite cell distribution, and they follow the same pattern of regeneration after cardiotoxin injury [104,105]. The latest research has shown that, utilizing a fibrin cellulous scaffold, myotubes can form and be used as a template. This template can be manipulated with BaCl_2_ injury and seeded with donor muscle stem cells, mimicking cell transplantation. It was also shown that the donor cells mediated muscle regeneration and their niche repopulation could be captured and analyzed [106]. Therefore, with the primary or iPSC-derived myogenic cells from a muscular dystrophy patient, 3D muscle models can be established to test cell engraftment and regeneration. This modeling system could be an alternative to animal testing and significantly reduce the number of animals being used. Considering animal welfare and the high failure rate in preclinical drug development, the FDA Modernization Act 2.0 was passed by the US government in December 2022 to encourage investigation of alternatives like cellular models to reduce the occurrence of animal testing [107]. A recent study was published to demonstrate the successful application of electro-physiological testing in 3D-engineered skeletal muscles. A therapeutic intervention was shown to alter the result. This kind of testing was previously only achievable through animal testing [108].

### 4.2. Animal Models

#### 4.2.1. Murine Models

Mouse models are widely used to study human diseases because the mouse has a small body size and short lifespan, and its genome is comparable to the human genome, which can be engineered for experimental purposes [92]. The clinical severity of each muscular disease depends not only on the functional loss of satellite cells and/or muscle fibers, but also on the degree of their dysregulated interaction with the different muscle-resident cell populations, e.g., the fibro-adipogenic progenitors (FAPs) [93,94], and with the immune cells recruited from circulation to support muscle regeneration [95,96]. Due to this inherent phenotypic complexity, the use of cellular models in vitro poses strong limitations in muscular disease studies. To overcome this obstacle, researchers have generated animal models for a number of muscular diseases to correctly identify the pathogenic relationship between the loss, or the re-activation, of a specific gene and the progression of the corresponding muscular disease, as well as to test the effectiveness of therapeutic treatments. In general, methods to generate mouse models for muscular diseases include naturally occurring, non-targeted, or chemically induced gene mutations and the disruption or modification of specific genes by classical gene targeting [97] or by TALEN or CRISPR/Cas9 technology [98,99]. In the following paragraphs, the most relevant mouse models for a set of muscular diseases are described.

##### Duchenne Muscular Dystrophy

The most used DMD mouse model is the naturally occurring Mdx mouse (C57BL/10ScSn-*Dmd^mdx^/J*). In the Mdx mouse, a C>T mutation in exon 23 of the X-chromosome *Dmd* gene results in the formation of a pre-mature stop codon and thus in the loss of the full-length dystrophin [109,110,111]. The Mdx mouse shows a 25% reduction in the normal lifespan when compared to the C57BL/6J mouse, and a mild, non-progressive DMD-like muscle phenotype, with some severe pathological signs after 15 months of age [112,113,114]. The mild dystrophic phenotype of the Mdx mouse is mainly caused by the compensatory increased level of the utrophin protein that may substitute dystrophin [115,116]. To overcome this, a mouse model null for utrophin was crossed with the Mdx mouse to generate the *Mdx/Utrn^−/−^* mouse, which shows a much more severe dystrophic phenotype than the Mdx mouse [117,118]. Since human DMD consists of a variety of mutations in different regions of the *Dmd* gene, additional Mdx mouse lines have been generated either by treating mice with N-ethylnitrosurea, which randomly induces point mutations in the genome, namely the *Mdx^2cv^*, *Mdx^3cv^*, *Mdx^4cv^*, and *Mdx^5cv^* mice [119,120], or by inducing exon deletions in the *Dmd* gene, e.g., the *Mdx52* mouse, through homologous recombination [121], and the humanized *hDMDdel45-mdx* mouse using CRISPR/Cas9 technology [122].

##### Facioscapulohumeral Muscular Dystrophy

FSHD is the third most prevalent muscular dystrophy [123]. In most of the cases, FSHD is autosomal dominant and is characterized by the progressive weakness and wasting of muscles of the face, scapular region, and upper arms, followed by muscles of the trunk and legs [124,125]. FSHD is caused by the ectopic expression of the DUX4 protein in the muscle. The *DUX4* gene maps in the D4Z4 macro-satellite repeat array of chromosome 4 (4q35), with each D4Z4 repeat containing an open reading frame (ORF) for the full-length *DUX4* [124,126,127,128]. DUX4 is a transcription factor active in early development, but absent in almost all adult tissues, due to the epigenetic silencing of the D4Z4 array [125,129]. There are two types of FSHD, 1 and 2; FSHD1 is caused by a reduced number of D4Z4 repeats, while FSHD2 is caused by the loss of functional epigenetic enzymes that normally silence the D4Z4 repeats, mainly the structural maintenance of chromosome flexible hinge domain containing 1 (*SMCHD1*) and DNA methyltransferase 3 beta (*DNMT3B*) genes [130,131].

Because the mouse genome is devoid of the D4Z4 array containing *DUX4* [132], but encodes only homologs of the human *DUX4* gene that, when overexpressed, do not induce a strong muscle necrosis [133], mouse models for FSHD were designed to express the human *DUX4* gene in a muscle-restricted manner from part of the human FSHD locus.

There are three main transgenic mouse models for FSHD. Firstly, the iDUX4pA mouse, in which a genomic fragment isolated from the terminal D4Z4 repeat of the human *DUX4* locus, including the DUX4 ORF and its downstream poly-adenylation sequence, has been integrated in the mouse X-chromosome under the transcriptional control of tetracycline-responsive elements (TREs) [134]. Muscle fiber-restricted *DUX4* transcription is driven through the expression of the reverse tetracycline-controlled transactivator (rtTA) protein under the control of the human actin alpha 1 skeletal muscle promoter [134]. When iDUX4pA mice are treated with doxycycline (dox), muscle fibers show signs of an FSHD-like myopathy, including widespread muscle necrosis, regeneration, inflammation, fibrosis, and reduced muscle force generation [95,135].

The second FSHD mouse model, the tamoxifen (TMX)-inducible Cre-DUX4 (TIC-DUX4) mouse, has been generated by inserting into the ROSA26 locus of the mouse a single human *DUX4* V5-tagged ORF (exon 1) plus its 3′ untranslated region (UTR), followed by the endogenous poly-A sequence (exon 3) and by a second poly-A sequence of the bovine growth hormone gene [136]. To avoid leaky production of DUX4, a neomycin-resistance cassette flanked by Lox-P sites was integrated upstream of the *DUX4* ORF [136]. To generate the TIC-DUX4 mouse, the ROSA26-DUX4 mouse was then crossed with the HSA-MCM mouse [137], in which a TMX-inducible Cre recombinase is selectively expressed in muscle fibers under the control of the human actin alpha 1 skeletal muscle promoter. Once activated by TMX, Cre excises the neomycin cassette, allowing DUX4 expression by the ROSA26 promoter. In the absence of TMX, TIC-DUX4 mice showed a low level of DUX4 expression only when old (>1.5 years), while TMX-treated TIC-DUX4 mice developed the typical histological and functional phenotype of an FSHD-like muscular disorder [136].

The third most used FSHD mouse model is the FLExDUX4 transgenic mouse [138]. This mouse was generated by inserting in the ROSA26 locus a non-tagged DUX4 ORF, including the endogenous 5′ and 3′ UTRs and the poly-A sequence. To impede leaky expression of *DUX4*, the genomic construct was integrated in reverse orientation, with a set of recombination sites that allows the flipping of the *DUX4* coding sequence upon Cre-mediated recombination, and thus the expression of *DUX4* driven by the ROSA26 promoter. Like the TIC-DUX4 mouse, the FLExDUX4 mouse is also crossed with the HSA-MCM mouse to express DUX4 only in muscle fibers [138]. When treated with TMX, the FLExDUX4/HSA-MCM mouse shows a strong FSHD-like phenotype with widespread muscle necrosis and regeneration, immune cell infiltration, and fibrosis. However, FLExDUX4 mice are affected by a substantial level of undesirable DUX4 expression in the absence of TMX, which results in a moderate FSHD-like myopathy [138,139].

##### Oculopharyngeal Muscular Dystrophy

Oculopharyngeal muscular dystrophy (OPMD) is a late-onset, mostly autosomal dominant muscular disorder, caused by the expansion of the trinucleotide repeat GCG in the 5′ end of the poly-A binding protein nuclear 1 (PABPN1) gene. The corresponding expansion of the alanine track in PABPN1 leads to the formation of insoluble aggregates of proteins and RNAs in the nuclei of muscle fibers, which results in damage to the eyelid and pharyngeal muscles [140]. There are several mouse models for OPMD. The most used are the A17.1 mouse, characterized by the muscle-restricted overexpression of the PABPN1 protein with a 17-alanine expansion [141,142], and a mouse in which the c-DNA of the mouse Pabpn1 gene, harboring a 17-alanine expansion, has been knocked in in one of the endogenous *Pabpn1* loci to obtain the expression of both expanded and normal Pabpn1 proteins in all the tissues [143]. Even if these mice show many clinical manifestations of OPMD, they also show some features that are not recognized in the skeletal muscle of the patient, like cell death [144].

##### Limb–Girdle Muscular Dystrophies

Limb–girdle muscular dystrophies (LGMDs) are a group of muscular disorders characterized by the progressive weakness of the shoulder and pelvic girdle muscle groups. LGMDs show genetic heterogeneity and pleiotropy with mutations affecting dozens of different genes [145]. Based on the specific pattern of inheritance, LGMDs are classified as LGMD1 (autosomal dominant) or LGMD2 (autosomal recessive), the predominant forms of LGMDs. Common forms of LGMD1 are LGMD1B, due to mutations in the *LMNA* gene encoding the lamin A/C protein, and LGMD1C, caused by mutations in the *CAV3* gene. The LGMD2 group displays sarcoglycanopathies, in which mutations occur in genes encoding most of the proteins of the sarcoglycan complex, SGCA, B, G, and D; LGMD2A (now LGMDR1) is caused by the loss of a functional Calpain 3 protein, and LGMD2B (now LGMDR2) is consequent to mutations in the *DYSF* gene. Several mouse models are in use to study LGMDs, both naturally occurring, such as the SJL/J mouse, harboring a splice-site mutation in the *Dysf* gene [146,147], and the *Capn3*^−/−^ [148], *Cav-3*^−/−^ [149], *Sgca^−/−^* (LGMD2D, now LGMDR3), *Sgcb^−/−^* (LGMD2E, now LGMDR4), *Sgcg^−/−^* (LGMD2C, now LGMDR5), and *Sgcd^−/−^* (LGMD2F, now LGMDR6) knock-out mice [150,151,152,153,154,155]. All these mouse models show a progressive myopathy resembling corresponding human LGMDs.

##### Myotonic Dystrophy

Myotonic dystrophy (DM) is the most common form of muscular dystrophy in the adult, with an autosomal dominant inheritance [156]. DM is characterized by pleiotropic symptoms, such as muscle weakness, atrophy, and myotonia, but also cardiomyopathy, brain abnormalities, and cognitive impairment [157]. DM type 1 (DM1) is caused by the expansion of the CTG trinucleotide repeat at the level of the 3′UTR of the DM protein kinase (*DMPK*) gene, while DM type 2 (DM2) is caused by the expansion of the CCTG sequence inside the first intron of the CCHC-type zinc finger nucleic acid binding protein (*CNBP*) gene [158,159]. In both DM1 and DM2,the expanded regions generate RNAs that accumulate inside the nucleus to form RNA foci that compromise the action of several RNA binding proteins (RBPs), and this may result in the production of toxic peptides through repeat-associated non-ATG (RAN) translation [157,159,160,161]. This evidence highlights the deep complexity of the etiology of this muscular disorder, which makes it difficult to generate the right experimental mouse models. Nevertheless, some DM1 and DM2 mouse models are available which were generated either by inserting expanded CTG inside the mouse *Dmpk* gene, or by creating transgenic mice using large human genomic sequences containing expanded CTG repeats [162,163,164,165,166]. Additional mouse models were created by ablating the genes of specific RBPs known to be sequestered by the nuclear RNA foci, like the muscleblind-like (MBNL) proteins, or by overexpressing members of the CUGBP Elav-like family (CELF) of proteins [167,168,169].

#### 4.2.2. Other Animal Models (Zebrafish, Dogs, Pigs)

In addition to mice models, zebrafish have been regularly used in muscle development research, as well as muscular dystrophy drug discovery. Although being evolutionally distant, zebrafish carry > 80% of human disease-related genes. Their muscle development, like that of other vertebrates, requires the expression of myogenic regulatory factors (e.g., MyoD and myogenin). In addition, Pax7+ satellite cells were found in adult zebrafish muscle. The main differences in muscle between zebrafish and other vertebrates are spatially separated fast and slow muscles and a somatic muscle fiber structure being retained throughout development. However, despite the differences, zebrafish muscle shares basic morphology and physiology with human muscle. Moreover, the zebrafish body is transparent and largely composed of skeletal muscle, which enables any muscle disease phenotype to be easily captured by imaging-based assays during early muscle development. There are a few zebrafish muscular dystrophy models available generated by either screening of naturally occurring or targeted mutations [170]. However, as the zebrafish genome has undergone an additional duplication, causing some genes to become polyploid, generating a null mutation is challenging. Therefore, some researchers utilized a morpholino antisense oligonucleotide to knock-down the mRNA of target genes. Currently, there are zebrafish models for DMD, LGMD, and CMD (congenital muscular dystrophies) available.

Although using small animal models is cost-effective, using larger animal models could allow better evaluation of diagnostic procedures and treatment strategies. However, due to their larger size and longer life cycle, a very limited number of muscular disorder models have been generated, with the majority of them focused on DMD. So far, there are two DMD-Mdx rat lines generated by TALENS, which targeted exon 23, and CRISPR, which targeted exons 3 and 16 simultaneously [171,172]. They were both reported to have more severe force decline, muscle fibrosis, and cardiac phenotypes than *Mdx* mice, and are more similar to DMD patients. Naturally occurring dystrophinopathies in dog models which display a similar progressive disease phenotype to DMD patients were used in preclinical trials for testing gene therapies. The most well-known model is golden retriever muscular dystrophy (GRMD), which was discovered in 1958, before the identification of the DMD gene [173]. GRMD shares similar clinical features to those of DMD patients, especially in regard to histopathological lesions. The more recently identified dog models include the border collie dog, which carries a naturally occurring single nucleotide deletion in exon 20 of the DMD gene [174], and canine X-linked muscular dystrophy in Japan (CXMDJ) [175]. Scientists also generated pig DMD models by cloning male pigs that lack exon 52 in the DMD gene; although the pigs display hallmarks of the DMD disease phenotype, they die before sexual maturity [176]. This was overcome by the generation of a female heterozygous version carrying the same DMD mutation. In recent years, great interest has been laid on developing an FSHD porcine model based on the research finding that porcine DUXC and human DUX4 activate a highly similar set of genes; therefore, generating a transgenic DUX4-expressing mini-pig could have the same disease implications for humans [177]. Another large animal model that has the closest genetic background is the non-human primate; although there is no naturally occurring DMD primate model, DMD rhesus monkeys were generated by targeted mutation in exon 4 and exon 46 using CRISPR-Cas9 [178]. By using large animal models, scientists could better understand the immune response after therapeutic interventions.

### 4.3. Animal Models for Myogenic Cell Transplantation Therapy

In preclinical trials of stem cell transplantation therapies, it has been a common practice to use animal models as hosts. The most important concern with this procedure is immune rejection. To overcome this, researchers have generated genetically modified animals that are immune-deficient or have a suppressed immune system due to use of immunosuppressant drugs. Immune-deficient mouse models have been the most cost-effective among the models. The development of the immune-deficient mouse model began in 1962 when nude mice were first generated and found to be able to accept human cell grafts [179]. SCID mice, which were generated in 1985, lack the enzyme DNA-dependent protein kinase catalytic subunit, preventing the maturation of B and T cells [180]. This model was only used for solid tumor engraftment, but not single cell suspension due to the existence of natural killer (NK) cells. Soon after, SCID nonobese diabetic (NOD-SCID) mice were derived by JAX laboratory to inhibit the NK cell and macrophage functions [181]. This strain of mice was widely used in the stem cell transplantation setting, but their short lifespan (6–8 months) has been a major limiting factor. In 1995, Shutlz backcrossed NOD-SCID mice to introduce a mutated interleukin (IL)-2rg, resulting in NOG and NSG expression, (NOD/SCID IL-2Rgnull), which prolonged the lifespan of these mice; however, these mice are not resistant to irradiation [182,183]. Later, Shultz et al. replaced SCID and IL2Rgnull with a defective recombination activation gene (RAG) to create irradiation-resistant NRG mice which have a lifespan of 37 weeks [184]. Since then, NSG and NRG mice have become the most commonly used mice strains in stem cell transplantation studies. The other strains include NOD/SCID/β2M, which allows reproducible engraftment but is limited in lifespan, and BNX mice, which lack T and B cells but have compensatory high levels of NK cells [185,186]. Some immunodeficient rat models are also available; one Sprague Dawley rat model has functional deletion of the RAG2 gene (SRG) [187,188]. A double knock-out model of RAG2 and IL2Rγ genes called the SDRG model was generated to further delete NK cells [189]. However, due to cost and availability, immunodeficient mouse models are more commonly used.

With the advancement of stem cell therapies to preclinical trials, there is an increasing need for disease animal models that are immunodeficient. Taking muscular dystrophy (MD) as an example, scientists have crossed MD mouse models with immunodeficient mice to generate a mouse model for xeno-transplantation of cells of human origin. One of the most commonly used models in preclinical trials is the NSG-*Mdx* 4^CV^ mouse [190]. Utilizing this model, scientists are able to transplant human myoblast single cell suspensions with the ability to regenerate into donor-derived muscle fibers. The significance of this model is that functional muscle improvement was detected after the cell transplantation, which was not achieved previously. Therefore, this model has been applied extensively in trials with stem cell therapy products [18,191,192]. Other immunodeficient models have been generated, for example Capn3KO-NSG mice, but no functional data have been reported from this model [193]. Despite more and more efforts going into developing immunodeficient muscular dystrophy mouse models, there is a significant shortage due to high cost. Alternatively, immunosuppressant drugs are adopted to reduce the immune response caused by xeno-transplantation of cells. The drugs that have been used in studies include glucocorticoid, cytostatics, calcineurin inhibitors, mTOR inhibitors, and antibodies [194]. However, due to their high costs and low efficiency, immunosuppressant drugs have not been widely applied in muscle cell transplantation in vivo studies.

Although mouse models have been developed for a variety of muscular dystrophies, the challenges for testing cell therapy in these models are (1) that the disease phenotypes in most of the available muscular dystrophy mouse models are milder than those in humans, which could increase the difficulty of measuring efficacy from transplanted cells; and (2) that very few mouse models have been crossed to the immunodeficient background, which is critical for cell transplantation. More development of appropriate mouse models is needed to advance the progress of the preclinical trial for iPSC-derived cell transplantation therapy.

## 5. Delivery Strategies

So far, in the majority of the studies, cells were delivered intramuscularly, which only allows the cells to be delivered locally to the muscle tissue. As muscle is a solid tissue and does not allow cells to migrate freely like blood, multiple injections are required to distribute the cells across the entire muscle. As muscular dystrophies affect multiple muscle tissues of patients, it is challenging to perform a large number of injections to deliver the cells to all affected muscle groups. Therefore, scientists have been seeking alternative methods of injection to deliver therapeutic cells more systematically. Gulio Cossu pioneered the systematic delivery into muscle tissue using mesangioblasts derived from adventitial pericytes of skeletal muscle that possess myogenic potency. His study demonstrated, by injecting the mesangioblasts into the intra-femoral arteries of DMD mouse and dog models, that mesangioblasts can migrate to the muscle tissue and form dystrophin-expressing donor-derived muscle fibers and ameliorate clinical symptoms [195,196]. This is based on the theory that mesangioblasts could cross the vessel wall due to the exposure of several migration-related cytokines. Despite the success in the animal models, the first clinical trial of the mesangioblasts in a single DMD patient only resulted in <1% engraftment and no restoration of muscle function [197]. Also initiated by Cossu’s lab, researchers reported that treatment of muscle cells with DLL4 and PDGFBB could convert skeletal myoblasts to adopt a pericyte fate. Later, it was shown that both treated primary satellite cells and hiPSC-derived MPCs could migrate into muscle after intra-femoral artery injection. Despite the low engraftment efficiency in the in vivo studies, they did cast light on the possibility of pursuing systematic delivery of myogenic cells [198,199]. Cell engineering to express surface proteins that interact with cytokines, facilitating cell extravasation and migration into muscle tissue, is a future direction in the field in hope of developing systemic delivery. However, overall, more research is needed to enable the systemic delivery of myogenic cells to effectively treat more muscle-related diseases.

## 6. Manufacturing Considerations for Clinically Compatible Cell Product

A dose for one patient in muscle stem cell transplantation trials can be in the range of billions of cells, which underlines the importance of having a robust, scalable manufacturing process in place to create an adequate amount of drug product. There are six phases in the manufacturing process of hiPSC-derived myogenic cells: tissue acquisition, hiPSC expansion, gene editing (if applicable), myogenic lineage specification, hiPSC-myogenic cell expansion, and final formulation (Figure 1B). First and foremost, in order to generate clinical-grade cells, all processes need to follow current good manufacturing practice (cGMP). Depending on the phase of the drug of interest, the FDA provides a Guidance for Industry on how to implement appropriate cGMP of the drug [200], which requires all material used for culturing to be manufactured in a cGMP-compliant facility and the cell culture process to be conducted following a cGMP-compliant protocol. The cGMP manufacturing of hiPSCs has been discussed in detail previously [201]. Here, we focus on the steps that are specific to hiPSC-derived myogenic cell therapy, which include myogenic lineage specification, myogenic cell expansion, and formulation.

### 6.1. Manufacturing Challenges for Myogenic Lineage Specification

As mentioned in Section 2.1, the typical transgenic-free myogenic lineage specification takes more than 25 days. Seeding density, induction compound concentration, and medium composition are critical during the first ten days of mesodermal lineage induction. In-process monitoring during these first ten days is critical to establish go/no-go decisions for manufacturing. However, due to the limited translational knowledge of this process, very few assays have been established. The commonly used method during these first ten days in culture is the detection of mesodermal marker (e.g., MSGN1 and TBX6) gene expression via qPCR or flow cytometry. However, there has not been a study that establishes the correlation of these genes and the final myogenic drug product quality. Developing reliable in-process monitoring assays remains a challenge, especially in an autologous setting.

### 6.2. Manufacturing Challenges for Cell Expansion

Traditionally, iPSCs and myogenic cells are cultured in 2D culture systems. Although 2D culture is common and appropriate, at times, for research and development, it is a limiting factor for increasing manufacturing capacity to produce target doses of the appropriate quality. The current options for expanding cell culture capacity, and thus hitting clinical dosing requirements, are two-fold, with strategies that employ scale-out (i.e., moving from culture flasks to other 2D cultures) or strategies that employ scale-up (i.e., moving from culture vessels to bioreactors or other 3D cultures) [202]. Comparing the two options, the latter method, which results in adapting the adherent cell culture to the suspension culture, is more viable for significantly improving the manufacturing capacity due to its cost-effectiveness and closed system methodology. One study showed that moving to a 3D system decreased open processing techniques to 0.02% of those required in a 2D system [203]. Scale-out and scale-up are not linear processes. In particular, for scale-up processing, emphasis must be placed on flow rate and the need for potential encapsulation to protect against sheer stress of the targeted cell type in the case of spinning/stir tank bioreactors [204].

Three-dimensional cell cultures in bioreactors are increasingly developed for large-scale cell production. Some research has been performed to develop suspension cultures for iPSC expansion, myogenic lineage specification, and myogenic cell expansion. Among these, the hiPSC expansion phase is the most studied process. The iPSC 3D culture methods that can be adapted to bioreactor cultures include self-aggregated spheroids [205], cells on microcarriers [206], and cells encapsulated in alginate [207]. Small- to large-scale hiPSC suspension culture systems are becoming commercially available. With 3D iPSC culture, iPSCs can be directly differentiated into 3D muscle organoids by switching media to achieve lineage specification (see Section 2.1.3), which simplifies the process in a 2D culture system requiring dissociation and replating. The direct 3D lineage specification from iPSCs has been performed with neuronal cells (poster: https://assets.thermofisher.com/TFS-Assets/BID/posters/3d-pluripotent-stem-cell-suspension-culture-medium-protocol-spheroid-nucleation-expansion-poster.pdf, accessed on 1 February 2024). However, as 3D iPSC culture is not widely employed in industry or academia, there are no published articles to date. Therefore, although 3D iPSC culture and differentiation are desirable for large-scale cultures, more research and development are needed.

Despite the exciting development of 3D cell culture, hiPSC-myogenic cell expansion has not been well adapted to the 3D culture system to date. There are few studies showing muscle cell lineage specification by forming 3D muscle organoids or spheroids that contain PAX7-expressing myogenic progenitor cells [29,33], but no purification was reported following the specification. For downstream hiPSC-derived myogenic cell expansion, one study showed that myoblasts can be cultured in a suspension as aggregates, but the majority of cells exit the cell cycle and do not proliferate [208]. Therefore, myogenic cell 3D expansion remains a challenge and further work on this front is warranted.

## 7. Future Challenges and Perspectives

Overall, there has been rapid progress in the use of iPSC-derived cells for therapy and several clinical trials are underway for the use of iPSC-derived progenitors to treat muscle as well as other disorders. Vertex and Viacyte have generated islets from pluripotent cells to treat diabetes and Cynata and others have generated MSC from iPSCs to treat a variety of diseases. Other groups, for example Orizuru, have initiated trials to treat eye disorders (see, e.g., Eyestem: www.eyestem.com, accessed on 1 February 2024) and cardiac disease (e.g., Orizuru: https://orizuru-therapeutics.com/en/ourbusiness/, accessed on 1 February 2024). Similarly, in the muscle field, several companies have begun the effort to use iPSC-derived cells. These include Myopax, Myogenica, and Vita Therapeutics.

The initial effort of most iPSC therapy companies has been spent on using autologous products to bypass the immune rejection of mismatched cells, but they have also made advances in developing hypoimmune cells, which could provide more cost-effective solutions for patients. Although still in its early stage, Universal Cells, Sana Biotechnology, and several academic groups have shown that it is possible to transplant cells engineered to avoid the immune reaction by either using overexpression of local immune suppressive agents or evading the immune surveillance system by deleting antigens recognized as foreign or overexpression of normal evasion “don’t eat me signals” like CD47 and PD-L1 that avoid macrophage and NK cell activation or tolerization signals such as IL-10, HLA G, and HLA E [209]. Clinical trials with such approaches are in progress, though not in the treatment of muscle disorders [210,211].

Another major direction in iPSC cell-based therapy is to not use the cells themselves but to use non-living derivatives of such cells. This includes nonnucleated cells, such as red blood cells, platelets, and exosomes derived from cells. Exosomes can be loaded by genetically engineering the cells to overproduce a biologic and direct its expression to an exosome or by physiochemical means [212,213,214]. Also, studies have shown that exosomes made by muscle cells have a greater benefit in treating rodent models of DMD than exosomes made by other cell types [215,216]. Therefore, exosomes could be produced from iPSC-derived muscle cells to enhance their delivery capacity. Further, the discovery of muscle-specific fusogenes such as Myomaker and Myomerger could allow them to be used as signals to direct fusion of exosomes generated from muscle cells [217,218,219]. The above forms the basis of a potential novel use of iPSC-derived skeletal progenitors to make muscle-specific exosomes that can be used for systemic and/or local delivery.

While these are all impressive advances, they are still in the early stage and there is an expectation that additional breakthroughs will occur that can make cell and gene therapy safe, effective, and reliable. One of the future directions that can drastically broaden the availability of iPSC-based therapy is manufacturing hypoimmune iPSC lines from healthy donors using modern gene-editing techniques. These cells can be further differentiated into muscle progenitor cells for a wide range of muscle-related disorders.

This is a very exciting time, the beginning of a flourishing decade for stem cell therapies. The FDA already predicts that approvals for biologics will exceed that of small molecules and the approval of cell therapies will exceed that of gene therapies, and one can only expect the gap to widen as progress in these fields continues.

## Figures and Tables

**Table 1:** Surface protein markers identified in different studies to isolate myogenic progenitor cells.

Study	Myogenic Progenitor Types	Post-Differentiation Day	Selection Marker	Purity after Selection
Magli et al., 2017 [38]	PAX7+ myogenic progenitor cells	Day 23	CD54high+	90% PAX7+ cells
Hicks et al., 2018 [27]	Myogenic cells that can fuse the most	Day 50	ERBB3+/NGFR+	High PAX7 and MYF5 expression by QPCR
Wu et al., 2018 [39]	MYF5+ myogenic progenitor cells	Day 15	CD10+/CD24-	Mainly MYF5+ cells
Choi et al., 2020 [40]	PAX7+ myogenic progenitor cells	Day 30	CD271high+	85 ± 3.24% PAX7+
Nalbandian et al., 2021 [28]	PAX7+ myogenic progenitor cells	Day 42	FGFR4+	98.5% PAX7+ cells

## Data Availability

This article does not report any data.

## Abbreviations

FGF—fibroblast growth factor; IGF—insulin growth factor; HGF—hepatocyte growth factor; hiPSC—human induced pluripotent stem cell; PSC—pluripotent stem cell; hPSC—human pluripotent stem cell; EGF—epidermal growth factor; TWEAK—tumor necrosis factor-like weak inducer of apoptosis; BSA—bovine serum albumin; DMD—Duchenne muscular dystrophy; MD—muscular dystrophy; BDNF—brain-derived neurotrophic factor; VEGF—vascular endothelial growth factor; PDGF—platelet-derived growth factor; IL-6—interleukin 6; LIF—leukemia inhibitory factor; iPSC—induced pluripotent stem cell; ZFNs—zinc finger nucleases; TALENs—transcription activator-like effector nucleases; cGMP—current good manufacturing practice; AAV—adeno-associated virus; ESCs—embryonic stem cells.
